# Human brain organoids and their ethical issues: Navigating the moral and social challenges between hype and underestimation

**DOI:** 10.1038/s44319-023-00007-3

**Published:** 2023-12-15

**Authors:** Andrea Lavazza, Alice Andrea Chinaia

**Affiliations:** 1 Department of Brain and Behavioral Sciences, University of Pavia, Piazza Botta 11, 27100 Pavia, Italy; 2 MInD-MoMiLab, IMT School for Advanced Studies, Piazza San Francesco 19, 55100 Lucca, Italy

**Keywords:** History & Philosophy of Science, Neuroscience, Science Policy & Publishing

## Abstract

Recent advancements in the field are forcing scientists and neuroethicists to balance opposite concerns. Some see no risks at all while some waive red flags.

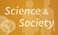

Since the field of neuroethics was established in the early 2000s, some scholars have proposed to distinguish between anticipatory and speculative neuroethics (Wexler, 2019). The former seeks to intercept potential ethical issues related to new scientific discoveries or a new technique or mode of intervention; the second, critics say, is an exercise of philosophers: imagining controversial situations that are only hypothetical and remain so. Ethical reflection about human brain organoid research seems to be characterized by this division.

Human brain organoids (HBOs) are biological entities grown in vitro from human stem cells that mimic aspects of brain function and organization. Given the obvious inability of obtaining in vivo research on human brains, scientists have been using cultured neuronal cells and animal models (Kelava and Lancaster, 2016; Pacitti et al, 2019) to unravel the mechanisms responsible for these and other pathological conditions. Yet, these methods retain crucial differences with real brains—three-dimensional organisation, species-specific differences, and so on—that make it very hard to study higher human brain functions. HBOs offer a novel method to address these shortcomings (Chiaradia and Lancaster, 2020).

Organoid research has recently turned ten years old and has shown itself to be one of the most promising areas of biomedical research. Since the first protocol for developing brain organoids was published in 2013 (Lancaster et al, 2013), researchers have used them to study a variety of normal and pathological conditions, effects of viruses on the human central nervous system or effects of toxic substances. Research efforts now aim to increase the complexity of brain organoids, for instance by reproducing nonneuronal cells, such as microglia, or growing vasculature. A recent publication also put forward the idea of harnessing the computational capacities of brain organoids to create what they call ‘organoid intelligence’ (OI; Smirnova et al, 2023). HBOs-focused research has also produced a large neuroethical literature about current and potential issues. In addition, at least one popular essay with autobiographical elements (*How to Grow a Human: Reprogramming Cells and Redesigning Life* by Philip Ball) and a novel in which brain organoids play a significant role (*The Revelations* by Erik Hoel) have been published.

## Risk of hype

As with other fields of biomedical research, the risk of hype is remarkably high. ‘Hype’ refers to an intense excitement or exaggerated enthusiasm surrounding a particular scientific idea, discovery or technology. It often involves widespread media coverage, public attention and anticipation, that create a buzz around the topic (Caulfield and Condit, 2012).

Consider, for example, human enhancement and cognitive enhancement in particular. There is broad consensus in the scientific community that the benefit gained from off-label use of molecules and noninvasive brain stimulation is rather modest, although not null. Many students and professionals, however, resort to those drugs and techniques in an attempt to improve their performance. Even if systematic research on cognitive enhancement cannot be conducted for safety and ethical reasons, one could contend that the interest in enhancement is potentially attributed not only to research that expressly seeks to assess the impacts of augmenting cognitive abilities but also the vast body of bioethics literature. This literature has envisioned scenarios involving individuals with superhuman capabilities and has engaged in extensive debates both in favor of and against the plausibility of such a prospect.

Nor should one forget the hype that surrounded embryonic stem cells, which were first isolated in 1998, and which are also the origin of some types of organoids. There was talk then of a medical revolution and the possibility of repairing damaged or diseased tissues and organs. Stem cell cures were advertised for a wide range of disorders, but no results were really achieved: “Yet today, more than two decades later, there are no treatments on the market based on these cells. Not one” (https://www.technologyreview.com/2023/08/09/1077580/embryonic-stem-cells-25-years-treatments).

## Hype around brain organoids

Is something similar happening to human brain organoids? Certainly, they manifest all the characteristics of hype. HBOs are *Groundbreaking Discoveries*: When researchers make significant breakthroughs or uncover something unexpected, it can generate a lot of excitement. Growing a 3D human brain model in a dish is certainly an exciting breakthrough. HBOs are *Emerging Technologies*: Advancements in technology can trigger hype, especially if they promise to solve major problems or improve people’s lives dramatically. Hopes that brain organoids will provide effective models for personalized treatment of some diseases are already becoming a reality. HBOs attract *Media and Public Attention*: Sometimes, scientific topics capture the media’s attention, leading to exaggerated coverage and a frenzy of public interest. This is certainly the case with brain organoids. HBOs create *High Expectations*: If a scientific idea or technology is surrounded by high expectations or promises of extraordinary outcomes, it can lead to a hype cycle. People might anticipate these advancements to have a swift and profound impact, even if the reality might be more gradual or nuanced. Finally, HBOs raise *Strong Ethical Concerns*: When a groundbreaking discovery gives rise to a powerful technology, it also creates fear of novelty and unforeseen implications along with the excitement. Adding to this phenomenon are the influential contributions of popular media, numerous neuroethicists and prominent public intellectuals. One can perhaps speak in this case of *moral hype*, linked to fears that research on brain organoids might cross some generally accepted ethical red lines, such as the manipulation of human beings. *Moral hype* thus does not concern an exaggerated expectation of a discovery or a strand of research, but it configures as an exaggerated fear related to experimentation in a specific biomedical field.

Moral hype thus does not concern an exaggerated expectation of a discovery or a strand of research, but it configures as an exaggerated fear related to experimentation in a specific biomedical field.

When faced with a hype scenario, especially *Strong Ethical Concerns*, we should rely on evidence rather than feelings and assumptions. Specifically, this relates to the potential emergence of some forms of consciousness among HBOs and the granting of moral status to these new entities. Therefore, we conducted the first (to our knowledge) research on the ethical orientations of a representative group of scientists working on HBOs (Lavazza and Chinaia, 2023). The research was based on semi-structured interviews with 21 senior and junior researchers and revealed overall a low level of ethical concern toward their main research object.

When faced with a hype scenario, especially *Strong Ethical Concerns*, we should rely on evidence rather than feelings and assumptions.

## Ethical concerns

The main striking result we observed was the fact that scientists did not seem concerned with the possibility of brain organoids becoming more than simple models—that is, they generally agreed that developing even a rudimentary form of sentience in the lab was not going to happen soon, nor potentially in a distant future. It follows that they did not acknowledge—with very few exceptions—the need of discussing the possibility of granting some kind of moral status or protection to such entities. To use the words of one of the participants, “when you actually work with these things, they look like something you take out of your nose. They are like little blobs; they really do not look like actual brains” (Lavazza and Chinaia, 2023).

It seems that the perspectives of researchers and some of the more critical neuroethicists are currently partially divergent and polarized. There are a few exceptions in the sample we surveyed and in the scientific community as well, but they do not seem to find any sides to their concerns.

The clear opinions of scientists over the issue of consciousness and moral status, however, does not mean that they undervalue the ethical discussion in general. In fact, some interviewees were aware of the possible issues related to other ethical aspects, such as informed consent in the case of HBOs grown starting from induced-pluripotent stem cells (iPSCs), or the problem of communicating the findings and the research to the lay public.

In line with what has been discussed at the beginning of this article, the possible overhyping of the technology was reported as something ethicists should pay attention to. This also relates to how one should call brain organoids, and if terms like ‘mini brains’ should be avoided (Bassil, 2023). A recent paper (Pașca et al, 2022) suggested to avoid such terms and prefer ones with a less science-fiction-like flavor. However, not all the researchers we interviewed agreed, suggesting that the risk of hype is counterbalanced by the risk of not making people understand what is going on in research.

Overall, the mainstream opinion over these issues seems to be going in the opposite direction of moral hype. In fact, the most recent recommendation from the National Academies of Sciences, Engineering, and Medicine (2021) did not identify any need for new rules in the field. Another recommendation of the International Society for Stem Cell Research (ISSCR) exempts HBOs from any specific ethical oversight (Lovell-Badge et al, 2021). Our interviewees align with these positions.

On the other hand, neuroethicists do report current or potential relevant ethical issues. Summing up such position is the dilemma formulated by Greely: “When we avoid unethical research by making living models of human brains, we may make our models so good that they themselves deserve some of the kinds of ethical and legal respect that have impeded brain research in human beings. If it looks like a human brain and acts like a human brain, at what point do we have to treat it like a human brain—or a human being?” (Greely, 2021).

Unlike Greely, many neuroethicists and bioethicists are extremely skeptical that there are relevant ethical issues associated with brain organoid research—and in particular, issues of the type that Hoppe and colleagues (2023) label “intrinsic”. They believe that it is extremely difficult to define what consciousness is and to study it in adult humans. Many theories of consciousness have been proposed over the years and a shared consensus is elusive (Zilio and Lavazza, 2023). Therefore, it seems even more unlikely that laboratory-grown HBOs can develop consciousness in the absence of interaction with the environment and that we could be able to detect any subjective sensations they could potentially experience. This leads, in line with what we have ascertained in our study, to downplay the debate concerning the moral status of human brain organoids and instead emphasize other ethical issues, such as informed consent and commercial exploitation.

## Science outpacing ethics

However, in seeking to mitigate the sensationalization of human brain organoids (Bassil, 2023) and addressing purported baseless ethical apprehensions, one should be cautious not to underestimate genuine or potential ethical dilemmas and valid public concerns. A noteworthy recent example serves as an illustration.

… in seeking to mitigate the sensationalization of human brain organoids and addressing baseless ethical apprehensions, one should be cautious not to underestimate genuine or potential ethical dilemmas and valid public concerns.

“We reasoned that the vascularization of neural organoids—while a necessary step to increase their size and complexity—does not mean that additional regulatory requirements are automatically warranted, even if organoids are transferred into the brains of laboratory animals. In the latter case, neuroscientists and regulators should focus on adhering to existing animal research welfare standards instead of worrying about the emergence of ‘human-like’ consciousness in animal models, because human neural organoids transplanted into animals lack the overall size, relevant cell types, and proper architecture to support cognitive abilities associated with higher-order thinking.” Thus argued a group of ethicists and scientists in a recent position paper, published on October 18, 2022 (Hyun et al, 2022).

Just six days earlier, a paper showed, for the first time, functional integration of human organoids transferred into the brains of laboratory animals. The authors “transplanted 3D hCO [human cerebral organoids] derived from hiPS cells [human induced-pluripotent stem cells] into the primary somatosensory cortex (S1) of immunodeficient rats at an early, plastic developmental stage. Neurons from transplanted hCO (t-hCO) undergo substantial maturation, receive thalamocortical and corticocortical inputs that are capable of evoking sensory responses and extend axonal projections into the rat brain that can drive reward-seeking behaviors (through optogenetics)” (Revah et al, 2022).

In the position paper (Hyun et al, 2022), the authors also wrote: “Our close conversations between neuroscientists and non-scientists led to the understanding that the public’s concerns about consciousness are biologically unfounded and will continue to remain so for the foreseeable future. […]. Although some scientific work by members of our working group has demonstrated the possibility of simple photosensitive reactions and motor outputs, neural organoids currently lack mature neural circuitry and have no systematic sensory input or effector output, making them unable to interact functionally with the environment in a complex manner. Thus, concerns about consciousness in neural organoids are currently unsupported. Importantly, for the responsible advancement of neuroscience, these scientific explanations must be disseminated to the wider public and policy makers to prevent uninformed, and therefore unjustified, barriers to research.”

Again, a few days earlier, on October 12, Kagan and colleagues published a paper demonstrating learning abilities of neurons grown in vitro (Kagan et al, 2022). The authors explained that “[i]n vitro neural networks from human or rodent origins are integrated with in silico computing via a high-density multielectrode array. Through electrophysiological stimulation and recording, cultures are embedded in a simulated game-world, mimicking the game Pong. Applying implications from the theory of active inference via the free energy principle, we find apparent learning within five minutes of real-time gameplay not observed in control conditions” (Kagan et al, 2022). In this case ‘sentience’ can be interpreted as “responsive to sensory impressions” through adaptive internal processes.

There is more. As previously mentioned, Hartung, Smirnova and colleagues proposed the term “organoid intelligence” (OI) to present “a collaborative program to implement the vision of a multidisciplinary field of OI. This aims to establish OI as a form of genuine biological computing that harnesses brain organoids. […]. We envision complex, networked interfaces whereby brain organoids are connected with real-world sensors and output devices, and ultimately with each other and with sensory organoids (e.g., retinal organoids), and are trained using biofeedback, big-data warehousing, and machine learning methods” (Smirnova et al, 2023).

Each incidence demonstrates that while some scientists and bioethicists were still reasoning about novel rules and deciding that none were necessary, other researchers were already pushing the frontier of science. It seems that there is a dichotomy between the provision that there are no problematic developments in HBOs research and the experiments being conducted in public and private laboratories. The sample of scientists surveyed in our interviews and other committees have firmly acknowledged that lab-grown brain organoids do not have intrinsic aspects related to consciousness that may call for a more in-depth inquiry and a revision of current guidelines and protocols. Research, however, has moved on since we conducted our interviews to novel possible applications of brain organoids that neither scientists nor neuroethicists had foreseen. It would be interesting to collect once more scientists’ opinions in the light of these novel proposals and findings and see if there is a shift of perspectives.

…while some scientists and bioethicists were still reasoning about novel rules and deciding that none were necessary, other researchers were already pushing the frontier of science.

While we leave these aspects to future research, we acknowledge that the scientific community has welcomed the discussion on other ethically relevant aspects—such as communication styles to avoid sensationalism (Bassil, 2023)—and has included such considerations in their daily practice (Pașca et al, 2022). In this vein, the potential risk to research posed by moral hype becomes evident when ethical considerations transcend academia and permeate the public sphere. The unpreparedness of the public to assess technical and ethical facets may result in succumbing to emotional reactions. Simultaneously, a complete dismissal of the issue as premature may overlook salient aspects and preclude supervisory and regulatory processes.

## Navigating emerging concerns

A judicious middle ground, only cursorily outlined here, would necessitate a conscientious recognition by the scientific community of the peculiar nature of brain organoids, alone and in combination with animal models. This would entail the formulation of guidelines to harmonize diverse sensitivities and requirements, subject to periodic updates. These guidelines should include considerations related to the potential manifestation of sentience, as well as aspects pertaining to informed consent and the commercial exploitation of organoid research.

In addition, scientists should engage the public to address concerns surrounding brain organoids. Insights from past experiences, particularly from the domain of genetics, where contentious situations arose without prior societal preparedness for emergent research paradigms, underscores the need for caution. Examples include the cloning of an animal and modifying the human germ line in two children. Even if cases of sentience in brain organoids may not be imminent and might not necessarily represent a breach of research ethics, anticipating these scenarios can help building the appropriate tools to navigate such a situation judiciously.

Yet, cases in which genetic modifications for different purposes are induced in human brain models might be closer. Recently, Legnini and colleagues (2023) tested “optogenetic perturbations in combination with spatial transcriptomics as a powerful technology to reprogram and study cell fates and tissue patterning in organoids.” However useful genetically modified human brain organoids may be, research should be accompanied by an appropriate ethical process. It is important to keep the dialogue between science and philosophy open, alive, and fruitful as it has been so far. Both research and ethics will benefit from it.

### Supplementary information

Peer Review File
