# Supplementary material for: Human brain organoids and their ethical issues: Navigating the moral and social challenges between hype and underestimation
Source: EMBO Rep. 2023 Dec 15;25(1):13–6. doi: 10.1038/s44319-023-00007-3 (PMC10897434; doi:10.1038/s44319-023-00007-3)
Supplement: Supplementary file 1 — Peer Review File [file 44319_2023_7_MOESM1_ESM.pdf]

EMBO reports

# Human brain organoids and their ethical issues

Allice Chinaia, Andrea Lavazza
**DOI: 10.15252/embr.202358149**

*Corresponding author(s): Andrea Lavazza (andrea.lavazza@unipv.it)*



## Review Timeline:

| | |
|---|---|
| Submission Date: | 9th Sep 23 |
| Accepted: | 10th Nov 23 |

*Editor: Holger Breithaupt*


## Transaction Report: This article was editorially reviewed.
